# Patient with lupus anticoagulant caused aPTT prolongation corrected with prednisolone treatment and later anticoagulation treatment due to chronic atrial fibrillation

**DOI:** 10.1002/ccr3.7284

**Published:** 2023-06-07

**Authors:** Marlena Frydrysiak, Paulina Pachniak, Anna Krysicka, Dariusz Moczulski

**Affiliations:** ^1^ Department of Internal Medicine and Nephrodiabetology Medical University of Lodz Lodz Poland

**Keywords:** aPTT prolongation, bleeding, case report, lupus anticoagulant

## Abstract

**Key Clinical Message:**

Lupus anticoagulant caused aPTT prolongation in rare case can cause bleeding tendency especially when combined with other hemostasis abnormalities. In such cases, aPTT value can be corrected by immunosuppressants within several days of treatment. When anticoagulation therapy is needed vitamin K antagonist are a good option for the initial treatment.

**Abstract:**

Lupus anticoagulant antibodies despite causing aPTT prolongation are commonly associated with increased risk of thrombosis. We present a rare case of patient when these autoantibodies resulted in dramatic aPTT prolongation and combined with associated thrombocytopenia resulted in minor bleeding events. In presented case treatment with oral steroids resulted in aPTT values correction followed by resolution of bleeding tendency within several days. Later, the patient developed chronic atrial fibrillation and was started on anticoagulation treatment initially with vitamin K antagonist without bleeding complications during follow‐up period. Corresponding changes in patient's aPTT time in a course of whole treatment is presented.

## 1 INTRODUCTION

Coagulation screening is performed routinely worldwide and can be used for individual bleeding risk assessment during a surgery or any other invasive procedure in higher‐risk patients. Isolated prolongation of activated partial thromboplastin time (APTT) is not common finding in general population and it is most often attributable to anticoagulant therapy^1^ or antiphospholipid antibodies. Less common reasons include deficiencies of a factor of the contact pathway, deficiencies of factors of the intrinsic and common pathways, von Willebrand disease, liver disease/vitamin K deficiency, hypofibrinogenemia, disseminated intravascular coagulation, and supercoumarin intoxication.^2^

Lupus anticoagulant is a class of antiphospholipid antibody causing a phospholipid‐dependent prolongation of the clotting time but is not usually associated with bleeding tendency but rather with increased risk of thrombosis and pregnancy morbidity. Lupus anticoagulant is regarded as rare case of bleeding^3^ with few examples that can be found in literature.^2^, ^4^ In special cases lupus anticoagulant caused aPTT prolongation can be also associated with acquired factor II deficiency causing lupus anticoagulant‐hypoprothrombinemia syndrome which is a rare disease predisposing to severe bleeding.^5^, ^6^

Atrial fibrillation is associated with an overall fivefold increased risk of ischemic stroke. However, this risk is not the same in all patients depending on the presence of specific stroke risk factors. Assessment of individual stroke risk in each patient with atrial fibrillation is crucial do identify those patients who need anticoagulation as stroke prevention treatment. CHA_2_DS_2_‐VASc (congestive heart failure, hypertension, age ≥ 75 years, diabetes mellitus, stroke, vascular disease, age 65–74 years, and sex category) score summarizes common stroke risk factors and is used for qualifying patients for stroke prevention treatment. Once the anticoagulation treatment is indicated, potential risk for bleeding also needs to be assessed. HAS‐BLED (uncontrolled hypertension, abnormal renal and/or hepatic function, stroke, bleeding history or predisposition, labile INR, elderly, drugs, or excessive alcohol drinking) score is a tool for predicting bleeding risk in such patients. Both stroke risk and bleeding risk stratification in patients with atrial fibrillation should be reassessed during treatment.^7^

## 2 CASE PRESENTATION

A 64‐year‐old woman was referred to our hospital by nephrologist due to observed nephrotic proteinuria. The patient had a history of systemic lupus erythematosus diagnosed 30 years ago with cutaneous lesions and arthritis treated with glucocorticosteroids and chloroquine without any documented further relapses. Her past medical history included chronic kidney disease stage G3/G4, gout, hypertension, complex heart defect correction—implantation of pericardial mitral bioprosthesis and tricuspid valve repair (2010), traffic accident (2014) followed up by skin grafts (2015, 2016), abdominal hernia repair (2016), and cholecystectomy.

Six months before, the patient had proteinuria of 2.5 g/day that increased to 6.75 g/day with stable serum creatinine level and periodically observed lower extremity oedema. Additionally the patient complained of several episodes of epistaxis. With the suspicion of lupus nephropathy the patient was admitted for kidney biopsy. She had slight lower extremity oedema and elevated blood pressure 195/96 mm Hg. Her laboratory tests revealed an isolated prolongation of aPTT to maximum value of 172.6 s (normal range 22.6–34 s), slight normochromic anemia (hemoglobin 11 g/dL), thrombocytopenia (115 × 10^3^/μL), elevated serum creatinine (170 μmol/L), ESR 75 mm/1 h (normal < 20), parathormone (147 ng/L), and TSH levels (7.670 mIU/L). Due to observed abnormalities in coagulation tests and tendency to extended bleeding, bruising and epistaxis patient was disqualified from kidney biopsy because of the high risk of bleeding. Further coagulation tests were performed. 1:1 mixing study showed no aPTT correction (107.9 s) what suggested a presence of anticoagulant or inhibitor of factor VIII. Serum levels of factor VIII, IX, XI, and XII were normal. Lupus anticoagulant test was positive at high titers along with positive anti‐dsDNA antibodies and ANA level of 1:1280. Anticardiolipin and anti‐beta 2‐glycoprotein I antibodies were found negative. Systemic lupus erythematosus relapse was diagnosed. After exclusion of infections sites the therapy with prednisone was started (60 mg of prednisolone per day). After 5 days of treatment the aPTT decreased to 100.8 s. The previously observed tendency to bleeding was resolved. After 30 days of treatment aPTT decreased to 40.5 s. Because diabetes was diagnosed after 2 months of prednisolone treatment, the dose was reduced and the therapy with mycophenolate mofetil was started.

Four months later the patient had to be admitted to the hospital because of symptoms of chronic heart failure, that is, general malaise, increasing lower extremity oedema, heart palpitations, and chest pain. Atrial fibrillation was diagnosed with rapid ventricular response 130–140/min. Increased natriuretic peptides were found and serum creatinine increased to 232 μmol/L. Echocardiography showed enlarged left atrium. Despite of implemented treatment atrial fibrillation did not convert to sinus rhythm. Due to implemented treatment with good control of heart rate the patient's condition improved significantly. Four months after the start of treatment aPTT was 50 s and proteinuria was 3.59 g/day. Because of the high risk of ischemic stroke associated with atrial fibrillation an anticoagulation treatment was started. Low molecular weight heparin was considered as a form of anticoagulation birding therapy but it was not implemented due to the patient's persistent thrombocytopenia and the high risk of heparin‐induced thrombocytopenia (HIT). Treatment with NOAC (non‐vitamin K antagonist oral anticoagulants) in turn would influence aPTT, which was used to control the treatment of SLE. Therefore, the initial anticoagulation treatment with acenocoumarol, a vitamin K antagonist (VKA), was started.

During the period of follow‐up, no bleeding was observed. Table 1 presents aPTT before treatment, during immunosuppressive therapy and during the therapy with acenocoumarol. Later, after the period of stable aPTT values the anticoagulation treatment was successfully conversed to NOAC.

**TABLE 1:** Changes of patient's aPTT during treatment.

Day of treatment	Therapeutic option	Patient's aPTT (sec) (normal 22–34)
0	Before treatment	172.6
3	Third day of prednisolone (60 mg)	151.1
5	Fifth day of prednisolone (60 mg)	100.8
30	One month of prednisolone (60 mg)	40.5
60	Two months of prednisolone (60 mg)	36.8
100	Two weeks of Mycophenolate Mofetil^a^ + GKS^b^	43.7
120	Two months of Mycophenolate Mofetil^a^ + GKS^b^	48.6
130	One week of VKA + Mycophenolate Mofetil^a^ + GKS^b^	41.5

^a^ Mycophenolate mofetil dose was 500 mg twice per day.

^b^ Prednisolone in reducing dosage.

## 3 DISCUSSION

In this case report, we present the case of patient with systemic lupus erythematosus with nephrotic syndrome and increased aPTT due to lupus anticoagulant. The observed bleeding could be associated with thrombocytopenia; however, it disappeared with decreasing of aPTT despite persisting thrombocytopenia. The immunosuppressive treatment with prednisolone corrected aPTT. In this case, the transfusion of serum would not improve aPTT as it was reflected in aPTT mixing study results. Because of increased aPTT, we were not able to do the kidney biopsy. Renal biopsy was crucial to diagnose a specific form and stage of lupus nephritis. Kidney biopsy should be done in all patients with lupus nephritis and nephrotic range proteinuria.^8^ Reduced prednisolone dose with mycophenolate mofetil treatment have the same impact on aPTT than treatment with high dose of prednisolone alone. Proteinuria decreased during the immunosuppressive therapy; however, no decreased of serum creatine during the follow‐up period was observed. New onset atrial fibrillation was observed in the patient during the therapy. Due to the high risk of stroke associated with atrial fibrillation, anticoagulant therapy was necessary. Choice of anticoagulation treatment was limited due to NOAC influence on aPTT assay^9^ and concern for the high risk of HIT development.^10^ The initiated vitamin K antagonist therapy did not interfere with aPTT and did not cause any bleeding during the follow‐up period.

## 4 CONCLUSION

Bleeding complications although rare have to be taken into account in patients with lupus anticoagulant. Increased aPTT can be observed in those patients combined with other coagulation disorders, that is, thrombocytopenia or acquired factor II deficiency. Anticoagulation treatment in SLE patients might be a challenge because of limited treatment options.

## AUTHOR CONTRIBUTIONS

**Marlena Frydrysiak:** Writing – original draft; writing – review and editing. **Paulina Pachniak:** Writing – review and editing. **Anna Krysicka:** Writing – review and editing. **Dariusz Moczulski:** Writing – review and editing.

## FUNDING INFORMATION

No sources of funding were declared for this study.

## CONFLICT OF INTEREST STATEMENT

The authors confirm that this article content has no conflict of interest.

## CONSENT

Written informed consent was obtained from the patient to publish this report in accordance with the journal's patient consent policy.

## Data Availability

The data that support the findings of this study are available on request from the corresponding author. The data are not publicly available due to privacy or ethical restrictions.
